# Chiricaspi (*Brunfelsia grandiflora*, Solanaceae), a Pharmacologically Promising Plant

**DOI:** 10.3390/plants7030067

**Published:** 2018-08-18

**Authors:** Carmen X. Luzuriaga-Quichimbo, Míriam Hernández del Barco, José Blanco-Salas, Carlos E. Cerón-Martínez, Trinidad Ruiz-Téllez

**Affiliations:** 1 Centro de Investigación Biomédica, Facultad de Ciencias de la Salud Eugenio Espejo, Universidad Tecnológica Equinoccial, Av. Mariscal Sucre y Mariana de Jesús, Quito 170527, Ecuador; luzuriaga.cx@gmail.com; 2 Department of Vegetal Biology, Ecology and Earth Science, Faculty of Sciences, University of Extremadura, 06071 Badajoz, Spain; mhernandye@alumnos.unex.es (M.H.d.B.); truiz@unex.es (T.R.-T.); 3 Herbario Alfredo Paredes, QAP, Universidad Central de Ecuador, Quito 170129, Ecuador; cecm57@yahoo.com

**Keywords:** activity, bioproduct, Brunfelsia, Amazonian, Ecuador, ethnobotanic, ayahuasca, validation, drug discovery, scopoletin

## Abstract

This study’s objective was to evaluate the rescued traditional knowledge about the chiricaspi (*Brunfelsia grandiflora* s.l.), obtained in an isolated Canelo-Kichwa Amazonian community in the Pastaza province (Ecuador). This approach demonstrates well the value of biodiversity conservation in an endangered ecoregion. The authors describe the ancestral practices that remain in force today. They validated them through bibliographic revisions in data megabases, which presented activity and chemical components. The authors also propose possible routes for the development of new bioproducts based on the plant. In silico research about new drug design based on traditional knowledge about this species can produce significant progress in specific areas of childbirth, anesthesiology, and neurology.

## 1. Introduction

Three species of the genus *Brunfelsia* (Solanaceae) used as an additive in the hallucinogenic “ayahuasca” drink have traditionally been very important plants for Ecuadorian indigenous Amazonian cultures such as the Kichwa of the East, Tsa’chi, Cofán, Secoya, Siona, Wao, and Shuar [1]. They are *Brunfelsia chiricaspi* Plowman, *Brunfelsia macrocarpa* Plowman, and *Brunfelsia grandiflora* D.Don, including ssp. *grandiflora* and ssp. *schultesii* Plowman in the variability range. Ancestral knowledge recognizes different applications or degrees of activity for each taxon. *B. grandiflora* is the one with the widest distribution area. It is well known as ornamental in Tropical America and is differentiated from its congeners by morphological characters related to floral and foliar size. This paper presents an ethnobotanical review of *B. grandiflora* ssp. *grandiflora* and *B. grandiflora* ssp. *schultesii*, in order to document its use, to offer arguments for its conservation, and to provide scientific evidence of its activity. The traditional knowledge rescued in a barely contacted Kichwa community in the province of Pastaza (Ecuador) is also presented. The authors describe the ancestral practices that remain in force today. They validated them through bibliographic revisions in data megabases, which presented activity and chemical components. The authors also propose possible routes for the development of new bioproducts based on the plant. In essence, this study’s fundamental objective was to conserve, document, and validate the traditional use of *Brunfelsia grandiflora*, as an element for innovation.

## 2. Results

### 2.1. Brunfelsia grandiflora: Botanical Description, Chorolorogy, Variability, and Names

**Figure 1:**
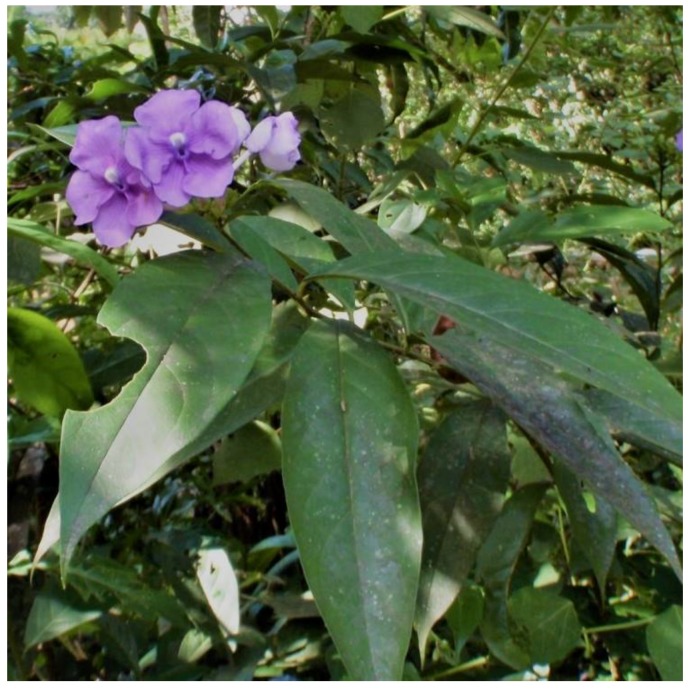
*Brunfelsia grandiflora* ssp. *grandiflora* in the Pakayaku rainforest, Ecuador. Photo credit: CX. Luzuriaga-Quichimbo (8 February, 2016).

*Brunfelsia grandiflora* (see Figure 1) is a small tree that was described by David Don in 1829 from material collected during the Ruiz and Pavon expeditions undertaken in Peru [2]. It has spread from Central America (Nicaragua, Costa Rica, USA) to the north of South America (Colombia, Brazil, Ecuador, Peru and Bolivia). There, it is well-known in cultivation as ornamental, exhibiting some exceptional forms. In the Amazon region, it is also cultivated for its narcotic and medicinal properties. It grows primarily at elevations of 650–2000 m, mainly on the eastern slopes of the Andes, in the region known as the montana (i.e., a humid, montane rainforest) [3].

The author of its monograph [3] gave the following detailed description: “Shrubs or small tree 1–6 (10) m tall. Trunk to 7 cm in diameter near base, much branched. Bark thin, roughish, light to dark brown. Branches slender, ascending or spreading, often subvirgate and arching, leafy, glabrous. Branchlets glabrous, rarely pubescent, green. Leaves 10–23 cm long, 3–8 cm wide, glabrous or sparingly pubescent at midrib; petiole 3–12 mm long. Inflorescence terminal and subterminal, simple or branched, dense or lax, the axis 5–45 mm long. Flowers 5-many, showy, scentless, violet fading to white with age, with rounded, white ring at mouth. Bracts 1–3 per flower, 1–5 (10) mm long, lanceolate to ovate, ciliolate, pubescent or glabrate, caducous. Pedicel 2–10 mm long, glabrous or with few sparse glandular hairs, becoming thicker and corky-verrucose in fruit. Calyx 9–13 mm long, tubular-campanulate, somewhat narrowed toward base, globose to obovate in bud, somewhat inflated or not so, glabrous, rarely punctate or sparsely glandular within, smooth or striate-nerved, light yellow-green to gray-green, firmly membranaceous to subcoriaceous, teeth 2–5 mm long, ovate-lanceolate, blunt to short acuminate, erect or incumbent, recurved slightly with age; calyx in fruit persistent, coriaceous, becoming corky-verrucose especially near base, often splitting on one or more sides. Corolla tube 30–40 mm long, 2.5–3.5 mm in diameter, 2.5–3.5 mm in diameter, the mouth 6–9 mm long; limb 35–52 mm in diameter, spreading. Stamens completely included in upper part of corolla tube; filaments thin, upper pair 4 mm long, lower pair 3 mm long, white; anthers 1–1.5 mm long, orbicular-reniform, light brown. Ovary 1.5–2 mm long, sessile, conical to ovoid, pale yellow; style slender, slightly dilated at apex; stigma about 1 mm long, briefly bifid, unequal, the up-per lobe somewhat larger, obtuse, green. Capsule 8–20 mm long, 8–20 mm in diameter, ovoid to subglobose, obtuse or apiculate at apex, smooth, nitid, dark green turning brownish, with corkypunctate or—verrucose outgrowths, pericarp thin, 0.3 mm thick, crustaceous, drying brittle, tardily dehiscent. Seeds 10–20, 5–7 mm long, 2–3 mm in diameter, variable in shape, ellipsoid to oblong, angular, dark reddish brown, reticulate-pitted. Embryo about 4 mm long; slightly curved, cotyledons 1.5 mm long, ovate-elliptic”.

**Figure 2:**
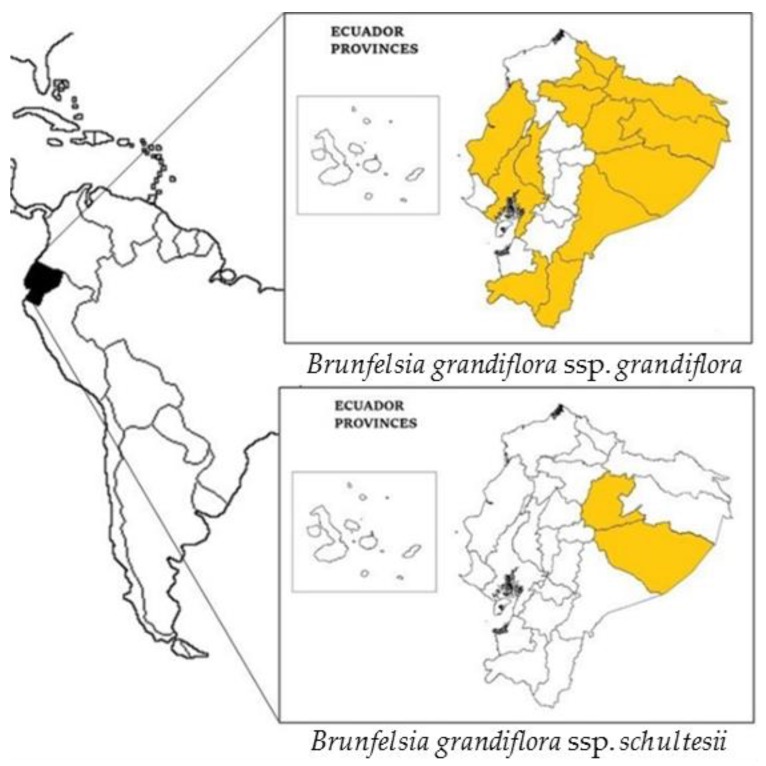
Distribution of *Brunfelsia grandiflora* s.l. in Ecuador.

*Brunfelsia grandiflora* ssp. *schultesii* Plowman, within the species’ variability and which has been collected [2] in Venezuela, Colombia, Brazil, Bolivia, Peru, and Ecuador (see Figure 2), was described by Plowman in *Bot. Mus. Leafl*. 23 (6): 259 (1973). It is characterized by its lower altitudinal preferences and smaller dimensions. The aforementioned author [3] described it in detail as follows: “Inflorescence variable, compact or lax. Pedicel 2–6 mm long. Calyx 5–10 mm long, teeth 1–3 mm long, triangular to triangular-ovate. Corolla tube 15–30 mm long, 1–2 mm in diameter, curved toward apex; limb 20–40 mm in diameter, spreading, mouth 3–5 mm long. Capsule 11–16 mm long, 10–16 mm in diameter”.

The species is referenced in Ecuador (see Figure 2) where it is called chiri kaspi, chiri wayusa, chiri wayusa pahu, chiri wayusa panka atu, urku chiri wayusa, wayra panka (Kichwa), uva silvestre (Spanish), i’shan ta’pe, luli ta’pe (Tsafi’ki), tsontimba’cco (A’ingae), jaija’o ujajai (Pai Coca), winemeawe (Wao Tededo), apaj, chirikiasip (Shuar Chicham), paiapia, and simora (unspecified language) [4].

### 2.2. Compilation of Brunfelsia grandiflora Ethnobotanical Uses

The Kichwa name chiricaspi (which means cold tree) refers to the chills and tingling sensations (”like rain in the ears”) that are felt after ingesting the bark. It is widely employed as a hallucinogen, often added to intensify the effect of narcotic drinks. Indigenous groups throughout the northwest Amazon use this plant to treat fever, rheumatism, and arthritis. It is said to act as a tonic over time, giving one strength and resistance to colds [3]. Ethnobotanical uses have been reported from different countries, for example, Bolivia [5], Brazil [6], Venezuela [7,8], Colombia [5,8,9], and Peru [8,10,11,12,13,14,15,16]. In Peru, the use of remedies based on this plant is traditionally associated with diet (refrain from eating pepper, i.e., ají, *Capsicum frutescens*), meat, and sexual relations [10,11]. Old thick roots are considered toxic, so only 2–3 roots approximately 1–1.5 cm diameter should be used [12]. Macerated roots are employed to combat rheumatism and syphilis. The leaves are also used to treat colds, arthritis, and snake bites. The bark is utilized against leishmaniasis. It is boiled to obtain a thick liquid that is applied to the affected areas [10,11,17]. In Colombia, both subspecies are commonly known as borrachero [3,9,12]. They are used in that country as an antirheumatic as well. Roots and, less frequently, the leaves are an admixture of ayahuasca. Prepared alone, this plant is used only when the shaman is faced with a particularly difficult or persistent problem, because the toxicity is known by local population. The most common preparation is making a tea from the roots and the bark. Cold water extraction can also be carried out, by shaving the bark from the roots and stems and then allowing them to soak. Alcohol mixture extractions have also been described [7,8]. In the Yabarana tribe (Venezuela), the leaves are routinely dried, crushed, mixed with tobacco and smoked [7,8].

Presented below (see Table 1) is a synthesis of the ethnobotanical knowledge about *Brunfelsia grandiflora* ssp. *grandiflora* and *B. grandiflora* ssp. *schultesii*, obtained from the indigenous communities of Ecuador based in bibliographic revisions [4,18] and our field prospections (see Table 2). Both subspecies seem to be used interchangeably in folk medicine, but ssp. *schultesii* is more widespread in the lowlands and is the form more likely to be employed.

### 2.3. Towards a Validation of the Pharmacological Action of B. grandiflora

**Figure 3:**
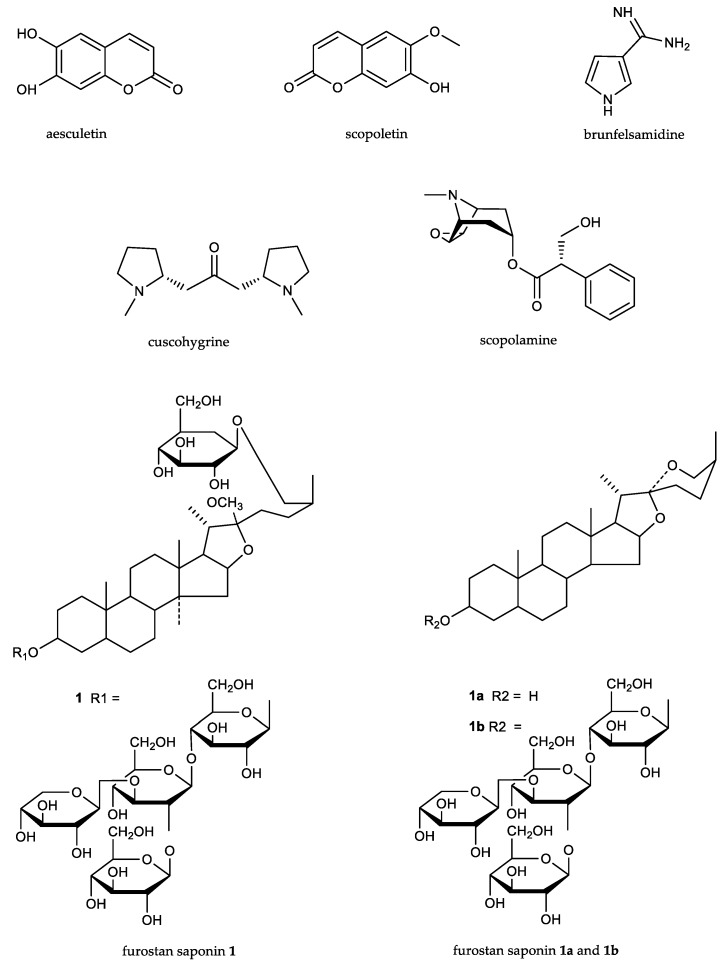
Chemical structures of the principal components of *Brunfelsia grandiflora* s.l.

The most important compounds found [5,7,12,19,20] in the leaves, stems, roots, and root bark are presented in Figure 3: coumarins, as aesculetin and scopoletin; a metilendiamine, as brunfelsamidine; unidentified alcaloids, as manacine and manaceine; tropanic alcaloids, as scopolamine; pirrolidinic alcaloids, as cuscohygrine; and steroidic saponins belonging to the furostan saponins type.

### 2.4. Experimental Studies on Activity

The principal evidence elements related to the physiological and pharmacological activities demonstrated by the experimental work carried out in the laboratory were selected and are summarized in Table 3. This table refers only to the activity of molecules that are contained in the species *B. grandiflora*. These activities have been tested and published in literature. The references that appear in the last column belong to the team author of the corresponding research for each case.

## 3. Discussion

*Brunfelsia grandiflora* is an interesting neotropical plant with worthy arguments for its conservation. It is locally cultivated as an ornamental decoration, as a personal decoration during traditional festivals, as an element of construction and even as a singular edible plant. However, its most unique use is cultural, linked to its administration by the local leaders of different ethnic groups. For example, it is used in the Ecuadorian Amazon, among the Cofán, to diagnose diseases or remove evil spirits; among the Shuar, to improve the aim or for inducing vomits; among the Siona to check paternity; among the Kichwa to improve luck during hunting. It has been used as a shamanic beverage in order to obtain knowledge about new medicines to treat diseases. It is ingested in rituals, is a relaxing, hypnotic, or hallucinogenic drug, and it is also prepared in the form of baths, along with orange leaves, caimito, achiote, grapefruit, and onion. As a medicinal plant it is used as an anti-flu medicine, to reduce fever and headache, to treat arthritis and rheumatism, wounds, burns, and even as a contraceptive. The subspecies *grandiflora* has been collected by botanists in the Bobonaza River basin [33], but the subspecies *schultesii* has not even been observed in the biggest province of Ecuador, Pastaza. Our survey revealed that the plant grows in the remote forest, where it was perfectly recognized by local inhabitants; it was currently being used and had a good reputation to combat rheumatism, arthritis, body pain, colds, and fever. Our fieldwork also provided three novel uses that had not been reported before: against stomach pain, as a larvicide against tupe, and as an accelerator of childbirth.

The validation of the pharmacological action of *B. grandiflora* can be supported by interrelating different information published in scientific literature. The anti-inflammatory effect of scopoletin [25,26], fully justifies the abovementioned uses against rheumatism, arthritis, body pain, colds, flu, fever, headache, joint and muscle pain, body blows, and discomfort. It can also explain its use by folk medicine as an anti-snake venom, and in cases of wounds and burns. The hallucinogenic and narcotic properties associated with symbolic cultural rituals clearly depend on the brain and nervous system activities that are mediated by brunfelsamidine [19], cuscohygrine [23], scopolamine [24], and even scopoletin [27,28,29], the last two sometimes exerting opposite effects. With respect to the traditional custom of associating this plant with increasing energy, wisdom, or marksmanship, the authors had to take into consideration the activity currently being studied in the furostan saponines (i.e., about their capacity to induce pro-sexual and androgenic enhancing effects [34]).

There are promising fields for innovation and scientific research in the future about this taxon. The antiproliferative effects of aesculetin [21,22] open the way in the field of oncology; brunfelsamidine and cuscohygrine [19,23], in the fields of anesthesiology and reanimation; and most relevantly, furostan saponins for the treatment of leishmania and other intracellular parasites that produce malaria [32]. The small molecular size of the main components presented in Table 2 make them very good candidates to be tested in the future “in silico” discovery of new drugs. Neurology seems to be one of the most current specialties, and their use in the childbirth process should also be addressed.

## 4. Materials and Methods

### 4.1. Study Area and Voucher Collection

The Kichwa community of Pakayaku (Bobonaza River, Pastaza, Ecuador) lies in an isolated region where bio- and ethnodiversity studies are still lacking. One of the authors (C.X.L.-Q.) was based in the Biological Station Pindo Mirador in the northern Bobonaza River basin (1° 27′ 09″ S, 78° 04′ 51″ W), and, since 2008, was charge of environmental monitoring and education programs involving the local population.

Plant collection permits were granted by the Ministry of the Environment, following the Convention of Biological Diversity rules [35]. Plant vouchers were deposited at the Herbarium José Alfredo Paredes, Universidad Central de Ecuador, Quito QAP Herbarium as “Ecuador. Pastaza: Sarayaku, Pakayaku, banks of the Bobonaza river, path to the lake by the house belonging to Mr. O. Aranda, 383 m, 01° 39′ 0.4″ S, 077° 35′ 53″ W, lowland evergreen forest, 9 February 2016, *C. X. Luzuriaga-Q & E. Gayas* sub subsp. *Grandiflora*: (QAP 93819); and subsp. *Schultesii* Plowman (QAP 93817).” The identification was revised by C. Cerón.

### 4.2. Ethnobotanical Survey

Collective written research consent was granted by Mrs. Luzmila Gayas, community president of the Assembly of Pakayaku. Prior oral individual consent was obtained from the persons taking part in the survey. Nagoya Protocol Rules were followed [35]. The investigation consisted of a series of planned house visits and walking routes accompanied by Kichwa interpreters and local inhabitants of Pakayaku. Interviews were semi-structured and included a series of open questions aimed to encourage discussion. All interviews were recorded. Ten knowledgeable elders of the Pakayaku community acted as informants and agreed to reveal their wisdom of the plant. The informants answered freely about several topics, namely the Kichwa common name, the part of the plant used, a description of the use, the harvest season, the storage (if any), the concoction, and the treatment target. After the field wok, the data were inserted into an MS Excel spreadsheet. All recorded uses were referred to standard classifications [4,18]. The data provided by the community (see Table 2) were compared with the existing ethnobotanical literature from Ecuador (see Table 1).

### 4.3. Scientific Validation

A bibliographic study was performed to provide scientific evidence for the medicinal uses of the plant. PRISMA statement reporting items were considered [36]. This included searching databases, removing duplicates, screening records, defining the eligibility criteria for articles, deciding about accessed and excluded articles, including selected articles, and studying the articles. The databases accessed were: Academic Search Complete, Agricola, Agris, Biosis, CAB Abstracts, Cochrane, Cybertesis, Dialnet, Directory of Open Access Journals, Embase, Espacenet, Google Academics, Google Patents, Medline, PubMed, Science Direct, Scopus, Teseo, and Web of Science by the Institute for Scientific Information (ISI). The selected citations are summarized in Table 3. A critical examination of Table 3 was the basis for the discussion of the results and the presentation of a specific and concrete conclusion.

## 5. Conclusions

This study presents the case of an Amazonian plant for which the approach adopted demonstrates well the value of biodiversity conservation in an endangered ecoregion. In silico new drug design, based on the traditional knowledge about chiricaspi, can produce significant progress in medical fields, such as neurology and anesthesiology.

## Figures and Tables

**Table 1:** Synthesis of the ethnobotanical knowledge about *Brunfelsia grandiflora* ssp. *grandiflora* D. Don and *B. grandiflora* ssp. *schultesii* Plowman * from the indigenous communities of Ecuador.

	Part	Formulation	Traditional Knowledge	Ethnic Group Ecuador Province
Human Consumption				
Edible Fruits/Sweet fruits	F	-	edible	Pichincha
Animal Feeding				
Edible Fruits/Sweet fruits	F *		avian food *	Wao * Orellana *
Building				
Houses, buildings, and agricultural facilities	S		building	Wao Napo
Industry and Crafts				
Cosmetics, perfumery, and cleaning	L	decoction or crushed and mixed with cold water	refreshing baths	Tsa’chi Pichincha
Tools and utensils (working, domestic, hunting, fishing, defense, etc.)	L, S		hunting tools	Kichwa del Oriente Orellana
Personal clothing and ornaments	F	clothing in festivals	personal adornment	Shuar Napo
Medicinal				
Conception, pregnancy, childbirth, and puerperium	L, S, R, B		contraconceptive	Kichwa del Oriente Napo
Respiratory system	B	a drop of juice resulting from crushing the bark is applied in the nose	flu	Cofán Sucumbíos
	R, L	coction		Kichwa del Oriente Napo
Musculature and skeleton	S	bark is removed	bloated and aching body	Kichwa del Oriente Orellana
	R	fumes *	rheumatism arthritis	Kichwa del Oriente Napo * Orellana
Skin and subcutaneous cellular tissue	L		burns	Kichwa del Oriente Orellana
	B *, L *	powder and infusion *	wounds and blows *	Kichwa del Oriente * Napo * Orellana *
Nervous system and mental disorders			headache *	Shuar Morona-Santiago
		fumes *		Kichwa del Oriente * Napo *
Symptoms and states of indefinite origin	L	infusion	body weakness	Kichwa del Oriente Pastaza
	L	powder and infusion *	chills and fever *	Kichwa del Oriente * Napo *, Orellana *
	B		chills and fever *	Secoya * Sucumbíos *
		infusion	healthy	Kichwa del Oriente Napo, Orellana
Social, Symbolic, and Ritualistic Uses				
Life Cycle Rituals	R, S		paternity test	Siona Sucumbíos
Rituals of uncertainty, protection, and affliction	R, L	infusion	induce vomiting for body purification	Shuar Napo
	L	infusion for bathing or drinking	improve luck during hunting, attract and tame animals	Kichwa del Oriente Napo, Orellana
	B	infusion for drinking	improve the aim of a blowpipe during hunts	Shuar Napo
		infusion for bathing, mixed with orange, onio, caimito, and achiote	protect against the “evil eye”	Kichwa del Oriente Orellana
	L	infusion	cause chills	Pastaza
		only for shamans (*)	obtain knowledge about new medicines (*)	Cofán * Sucumbíos
Hallucinogen, narcotic, and smoking	B	infusion, only for curacas	“become a tiger”	Cofán Sucumbíos
				Shuar Napo
	S	crushed with cold water, the shaman swallows it	disease diagnosis and to remove evil spirits from the body	Cofán Sucumbíos Shuar Napo
	B, R, S, L	drinks, sometimes mixed with *Banisteriosis caapi* to intensify the effect	hallucinogen used in rituals	Secoya *, Shuar Kichwa del Oriente Orellana, Sucumbíos Napo, Zamora Chinchipe

Part used: B, bark; R, root; S, stem; L, leaf.

**Table 2:** Specific medical and cultural uses of *B. grandiflora* ssp. *grandiflora* and *B. grandiflora* ssp. *schultesii* * in Pakayaku (Pastaza, Ecuador).

	Part	Formulation	Traditional Knowledge
Medicinal			
Digestive system	L	a handful of leaves is cooked, from thirty minutes to an hour, in two liters of water; it is taken in small glasses on an empty stomach	stomach pain and diarrhea ###
Conception, pregnancy, childbirth, and puerperium	B	scrape, mix it with a small glass of warm water, let it hover, and drink it	childbirth ###
Insect or other animal bites	B	scrape a piece and put it on the injury (tupe); repeat it when the product becomes dry, until the worm gets out	against tupe* ###*
Musculature and skeleton	R *	small roots scraped and tied with a rag or bandage and placed twice a day on the affected part *	aching body *
Skin and subcutaneous cellular tissue	B *	the bark is grated, deposited on a rag or bandage and tied to the area that has been hit*	body blows *
	L *	the leaf is crushed, placed on the affected area, tied, and then the patient falls asleep; the process must be repeated as many times as possible*	skin tumors *
Social, Symbolic, and Ritualistic Uses			
Rituals of uncertainty, protection, and affliction	B	it is prepared in a pot and a glassful is swallowed; during the treatment, a diet must be followed (refrain from taking salt or chili, *Capsicum frutescens*), nor should you stay near the candle	improve men’s energy when going into the forest
	B	the bark is grated, mixed in a medium recipient called pilchi, with water and taken at midnight; they also inform the authors that consuming this brew produced a lot of cold and chills	ensure strength blowing the blow pump during hunts

Part used: B, bark; R, root; S, stem; L, leaf. ### Recovered.

**Table 3:** Biological activity of some chemical compounds present in *Brunfelsia grandiflora.*

Molecules	Activity	References
aesculetin	inhibition of cancer cell migration	[21]
	antileukemia	[22]
brunfelsamidine	convulsant, affects serotonin levels	[19]
cuscohygrine	short-term ganglion-blocking	[23]
scopolamine	anticholinergic	[24]
scopoletin	anti-inflammatory by cytokine suppression	[25,26]
	spasmolytic by inhibition of calcium moving	[27]
	cholinergic in vivo rat brain	[28]
	blood pressure regulator	[29]
	hepatic steatosis protector by enzymatic inhibition	[30]
	antifungal	[29]
	antibacterial	[31]
saponins	antileishmania	[32]
